# Integrated Disaster Risk Management (IDRM): Elements to Advance Its Study and Assessment

**DOI:** 10.1007/s13753-023-00490-1

**Published:** 2023-05-23

**Authors:** Vicente Sandoval, Martin Voss, Verena Flörchinger, Stephan Lorenz, Parisa Jafari

**Affiliations:** Disaster Research Unit, Freie Universität Berlin, 12165 Berlin, Germany

**Keywords:** Integrated disaster risk management, Literature review, Proto-indicators, Sustainable development

## Abstract

This study analyzed the international key literature on integrated disaster risk management (IDRM), considering it a dynamic sociocultural process subjected to the historical process of social formation, offering a closer look at the concept while exploring conceptual elements and ideas to advance IDRM in both national and international contexts. Methodologically, the study adopted a literature review strategy, following the Preferred Reporting Items for Systematic Reviews and Meta-Analyses (PRISMA) approach, combined with qualitative content analysis. This article examines the history of IDRM, discusses current challenges for implementation, looks at some experiences, and proposes avenues for further research. Some findings point out the lack of an overarching IDRM approach, which is characterized by a rather disperse set of ideas and experiences concerning what IDRM is and how it should be operationalized, thereby revealing the need for a more comprehensive theory and methodologies to further advance it. Other findings highlight that IDRM encompasses different kinds and levels of “integrations” that go from internal (that is, disaster risk reduction and management domains) to external (that is, all societal processes such as sustainable development), including temporal and spatial integrations. Hence, we are talking about a multidimensional integration of disaster risk management. In this regard, the article proposes four dimensions for integration: sectoral, spatial/hierarchical, temporal, and externally with other cross-cutting societal challenges, especially climate change and sustainable development. These dimensions cover 29 ideas for indicators or “proto-indicators” to guide the discussion, exploration, and analysis of IDRM in specific contexts.

## Introduction

We know, so far, that the idea of an integrated disaster risk management (IDRM) has been around for at least 3 decades. Starting from the 1990s, conversations on integration and disaster risk management (DRM) have been intertwined with concepts such as sustainability and climate change. Nevertheless, conceptualizing IDRM has been elusive partly because it has never taken a central place in the disaster discourse and partly because “integration” tends to mean a lot of things to a lot of people and fields, from system research to sociology and anthropology.

In this work, we investigate IDRM from an international perspective and analysis in national contexts, adopting a politico-economic and social constructionist approach. By conducting a literature review and analyzing key definitions from selected works, we dig into how the concept of integration has emerged, and we ask what elements of a DRM system are necessary to consider it “integrated.” Some of these questions, such as where did the idea of IDRM come from, what does IDRM really mean, and how can we assess or evaluate “integration” in a national context, have guided this research.

The starting point is a basic yet significant interpretation of DRM. According to widely used international definitions, DRM “is the application of disaster risk reduction policies and strategies to prevent new risk, reduce existing risk and manage residual risk, contributing to the strengthening of resilience and reduction of disaster losses” (UNDRR 2022c). Although useful for some contexts, this definition leaves aside key elements that later will be fundamental to better understand “integration” in the context of DRM. This is the case of understanding DRM as a social product intrinsically tied to the way that different groups define risk and their means to “manage” it or reduce it at a specific time in history. This relational approach entails that DRM is a dynamic sociocultural process subjected to the historical process of social formation, routinization of everyday life, and institutionalization, implying that it is embedded in and is the result of societal relations and processes that are historically defined (Voss and Dittmer 2016). A systemic view of disaster risk formation/definition, management, and reduction considers that DRM is largely affected by societal everyday life experiences and definitions, including processes of power relations, division of labor, and class, among others. This relational approach is also linked to the systemic nature of risk (Voss and Dittmer 2016; Kelman 2020; Murray et al. 2021) where disaster risk is associated with cascading impacts that spread within and across systems and sectors (for example, urban settings, ecosystems, health, food supply, and critical infrastructure) through the movements of capital, goods, information, and people across regions and countries (Sillmann et al. 2022). The impacts of the COVID-19 pandemic, climate change, and more recently the war in Ukraine clearly show how the challenges of addressing risk in an interconnected and interdependent world go beyond traditional notions of DRM and risk governance (Merkes et al. 2020). These crises have also demonstrated the need to better understand “compound risk”—amplified by underlying vulnerabilities—and “cascading impacts,” as well as the political and societal responses to disasters. Consequently, addressing these complexities from a systemic viewpoint will also require integrating different cultural and politico-economic perspectives and fostering system thinking while implementing key intergovernmental agendas, such as the New Urban Agenda, the Paris Agreement, the Sendai Framework, and the Sustainable Development Goals (Sillmann et al. 2022).

Along the literature review, we analyzed different IDRM ideas and experiences—especially from China, Japan, and Mexico—to later explore potential indicator candidates or “proto-indicators” as proposed by Czúcz et al. (2021). These proto-indicators are considered entry points for further discussions on IDRM. In total, 29 proto-indicators were found, grouped in three meta-categories that relate to different kinds of integrations detected in our study: sectoral, spatial/hierarchical, and temporal. We consider that a prospective analysis may help to guide the discussion and support strategic planning for IDRM implementation in national and international contexts. Finally, the reflections collected in this study may help to guide upcoming discussions and implementations of IDRM, as expressed in the Sendai Framework for Disaster Risk Reduction 2015−2030 (UNDRR 2015), and especially in a context where integration between complementary global agendas is crucial.

## Methodology

**Fig. 1:**
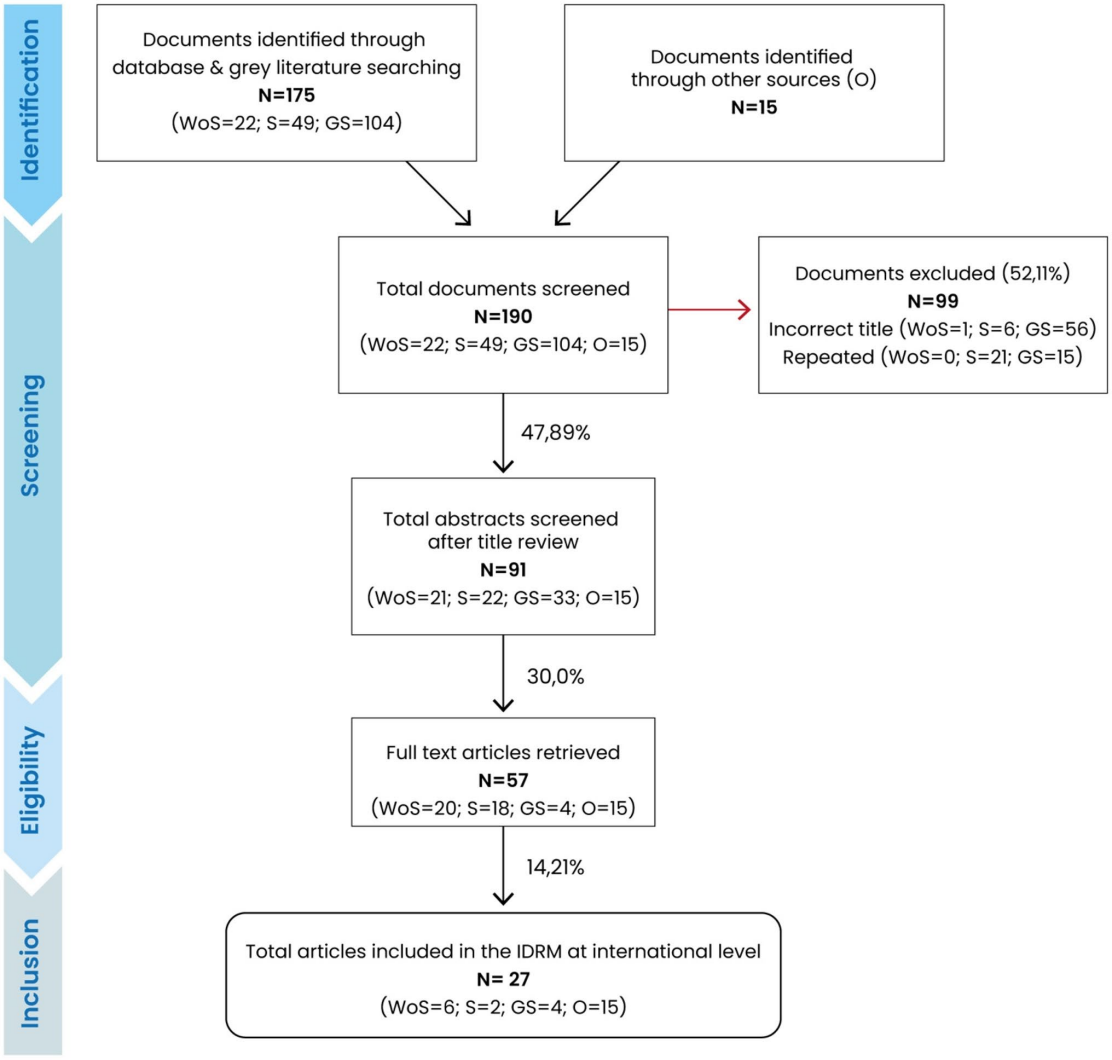
Flow of the literature search on integrated disaster risk management (IDRM). *WoS* Web of Science Core Collection, *S* Scopus, *GS* Google Scholar, *O* Other sources

In order to examine different approaches on IDRM at an international level, we adopted a literature review strategy following a Preferred Reporting Items for Systematic Reviews and Meta-Analyses (PRISMA) approach (Page et al. 2021) to existing published material (Fig. 1). We identified a list of databases (both scientific and grey) to find articles and publications about the integrated management of disasters and risks. The databases selected were Web of Science, Scopus, and Google Scholar; the Google search engine was used to look for other relevant websites. The second step entailed the definition of inclusion search criteria, screening titles and abstracts with related terms (that is, integrated disaster risk management) or related concepts such as “integrated management” and “disaster risk.” Articles were excluded if they were editorials, opinions, or commentaries without any substantial evidence independent of the study design and if the content was unrelated to the topics or not available on the Internet (see Table 1). Additionally, we reviewed the databases of several international organizations, such as the United Nations Office for Disaster Risk Reduction (UNDRR), the German Agency for International Cooperation (GIZ), and the Integrated Disaster Risk Management (IDRiM) Society, among others, as well as the reference lists (cross-referencing) of the articles judged to be relevant to the topic of interest.

**Table 1:** Search strategy

Source	Search strategy (Full Results)
Web of science (WoS) core collection	*TOPIC [Title-Abs-Key]—Timeframe: none* “integrated disaster risk management” (13) “disaster risk” AND “integrated management” (9)
Scopus (S)	*TITLE-ABS-KEY—Timeframe: none* “integrated disaster risk management” (35) “disaster risk” AND “integrated management” (14)
Google scholar (GS)	*Allintitle-[all-text]-sorted by date* allintitle: “integrated disaster risk management” (92) allintitle: “disaster risk” AND “integrated management” (12)

As the content structure of the selected documents tends to differ (that is, academic and grey literature), we adopted a qualitative approach to content analysis (Corbin and Strauss 2014). Documents were assessed on MaxQDA 22.0.1, looking at potential definitions and description of empirical cases related to IDRM while considering a historical perspective (that is, how the idea of IDRM has evolved).

We developed content categories to help us define IDRM at an international level in terms of what elements are necessary to consider a DRM system integrated, for example, multi-hazards approach to risk management and consideration of inter- and transdisciplinary research on risk, among others. Based on this, we briefly explored some results of the literature review with documented experiences from China, Japan, and Mexico. Finally, we refined these content categories into “ideas for indicators” (or proto-indicators) using Czúcz et al.’s (2021) approach as they may work to explore and analyze IDRM in specific national contexts.

## Results: Integrated Disaster Risk Management from an International Perspective—From “Adding” to “Doing Differently”

In 1987, the General Assembly of the United Nations designated the 1990s as the International Decade of Natural Disaster Reduction (IDNDR), and since then there have been at least three world conferences on disaster risk reduction (DRR) that have resulted in three major frameworks for action: the Yokohama Strategy and Plan of Action for a Safer World 1994–2005, the Hyogo Framework for Action 2005–2015, and the Sendai Framework for Disaster Risk Reduction 2015–2030. These frameworks have brought together ideas, concepts, and actors around common topics, such as better hazards monitoring, reduction of vulnerability and disaster impacts, and strengthening resilience. For instance, the Yokohama Strategy highlighted gaps and challenges on early warning systems; then the Hyogo Framework underlined the need for enhancing resilience while stressing the importance of proactive DRM over reactive measures. Finally, the Sendai Framework looks at disaster from a more systematic point of view, considering the underlying factors that influence the creation and reduction of risks, such as governance and those risks related to sustainable development. Other aspects have remained a constant challenge during these three decades. This is the case of IDRM.

In the 1994 Yokohama Strategy, the concept of integrated management of disaster risks pointed to a better strategy to achieve goals and objectives. It assumed that disaster response approaches alone are not sufficient to reduce disasters and risks; on the contrary, it presented disaster prevention as a fundamental element: “it contributes to lasting improvement in safety and is essential to integrated disaster management” (UN-IDNDR 1994, p. 2). Interestingly, the idea of “integration” that influenced the Yokohama Strategy can be tracked back to the 1992 Rio Declaration (UNCED 1992), where the integration of environmental and development concerns was the paramount goal.

**Fig. 2:**
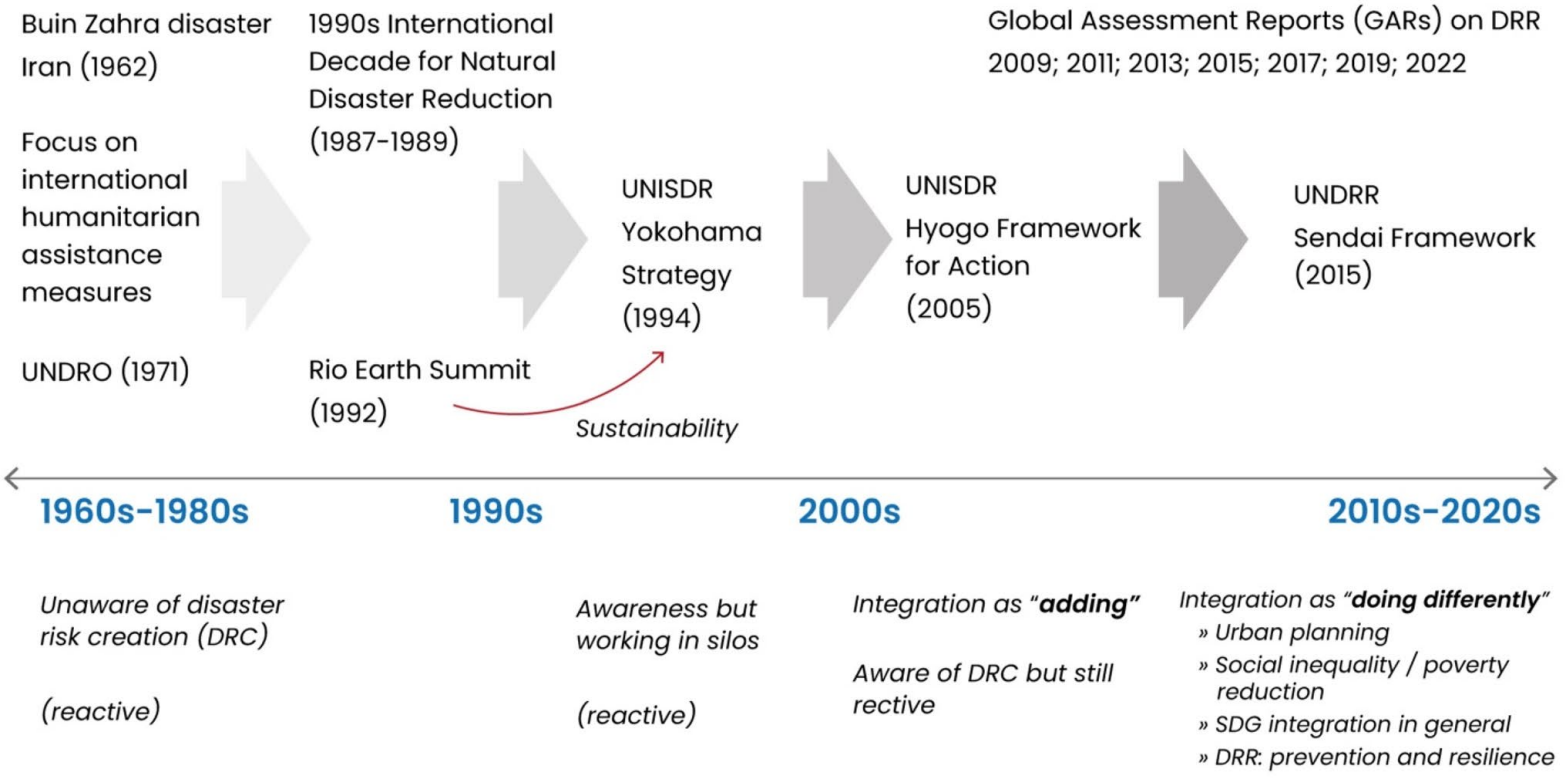
Historical development of the integrated disaster risk management (IDRM) concept

Figure 2 illustrates landmarks in the historical development of IDRM ideas. Between the 1960s and 1980s, the international debate tended to concern how to provide coordinated and efficient humanitarian assistance after disasters. This period started with the international efforts to provide humanitarian assistance to Iran after the Buin Zahra disaster in 1962, and it culminated with the creation of the United Nations Disaster Relief Office (UNDRO) in 1971. This period is characterized by a “reactive” approach to disaster as well as a lack of awareness about the underlying causes (referred to in Fig. 2 as disaster risk creation (DRC)).

During the 1990s, the foundations for IDRM were laid by incorporating the idea of sustainability and development (derived from the 1992 Rio Earth Summit negotiation) into the discourse of disaster and risk reduction. Although global objectives still pointed to a reactive approach in 1994, there was a growing awareness of DRC and the necessity to “integrate” different actors and development processes. This type of integration is defined by Wisner (2011) as “integration by adding,” and it is characterized by a growing institutionalization of DRM with a multi-disciplinary approach.

By 2002, the Johannesburg Plan on sustainable development requested actions under the chapeau: “An integrated, multi-hazard, inclusive approach to address vulnerability, risk, assessment and disaster management, including prevention, mitigation, preparedness, response and recovery, is an essential element of a safer world in the twentyfirst century” (United Nations 2002, p. 20). This supports a holistic approach that integrates DRM and DRR efforts and principles into sustainable development processes. However, it was the Hyogo Framework (UNISDR 2005) that prioritized the multi-sectoral integration of DRR among relevant actors beyond the conventional domains of DRM (that is, emergency response, early warning systems, and so on) and stressed the need to integrate DRR into development policies at all levels of government. For Zhang et al. (2004), IDRM means the management of all types of disasters and all phases of disaster management, focusing on hazard and vulnerability, including the underlying causes of risks, and emphasizing a multi-level, multi-sectoral, multi-disciplinary coordination among stakeholders. In Zhang et al.’s words, IDRM “represents a kind of philosophy and concept, a kind of basic system arrangement, a kind of integrated flow, a kind of mode and scientific method and a kind of future trend of disaster management” (Zhang et al. 2004, p. 115).

Likewise, the Sendai Framework deepens these notions of integration by focusing on reduced vulnerabilities and exposure and strengthening resilience. For example, recent mid-term reviews point out “risk-informed sustainable development” to stress the linkages between DRR and development (UNDRR 2020; Sillmann et al. 2022), such as the underlying causes of inequality, poverty, and other forms of vulnerabilities. The Sendai Framework asserts that the realization of DRR requires “the implementation of integrated and inclusive economic, structural, legal, social, health, cultural, educational, environmental, technological, political and institutional measures that prevent and reduce hazard exposure and vulnerability to disaster, increase preparedness for response and recovery, and thus strengthen resilience” (UNDRR 2015, p. 12). This aspirational goal highlights the relevance of vertical and horizontal integration through DRR and DRM policies articulated from international to local levels and among different actors and cross-cutting societal issues, such as the Sustainable Development Goals (SDGs) and climate change. Unfortunately, some studies have pointed out the Sendai Framework’s lack of concrete connections with these societal issues and processes: “despite the rhetoric of vulnerability, the measurement of progress towards DRR remains event/hazard-centric” (Chmutina et al. 2021, p. 786).

Wisner (2011, p. 2) refers to this overall integrative approach as “doing things differently” and calls for development in a “risk aware manner.” The latter means “integrating” the awareness of disaster risk creation and reduction into other aspects of development. For instance, the World Bank’s Global Facility for Disaster Reduction and Recovery (GFDRR 2009) has shown how sectors such as water, agriculture, and housing have been made risk-sensitive in a number of countries.

In this sense, integrated DRM can be seen as a process that involves the whole society and its activities and relations on all scales. A relational approach—in Holling’s (1973) ecological sense—is particularly useful here. Complex systems where nature and society are interconnected and interdependent do not behave deterministically, predictably, and systematically, but “systemically,” with complex feedbacks that cannot be conclusively described and that “cross” multiple scales, temporally and spatially (Voss and Dittmer 2016). Then, a systemic understanding of integrated DRM should start by considering the general conditions of the interplay of stabilizing and destabilizing variables that lead to a system maintaining a certain coherence and structure despite rapid and radical variation of internal elements and the surrounding environment (Voss and Dittmer 2016). In other words, “change” on multiple scales should be considered the norm, with periods of slow, faster, and suddenly radical changes, whereby different spatial and temporal scales interact (Gunderson and Holling 2002). This is the context for IDRM.

It is with this background that Shi (2012, p. 140) emphasized the importance of governance in IDRM and of mapping the roles of the various actors to understand the complexity of integration: “The range of actors and the design of the processes vary a great deal depending on the specifics of the political systems as well as the socioeconomic and cultural contexts.” However, examining IDRM actors and their relationships, as well as structures and functionality (formal or informal) of IDRM processes is methodologically challenging. Analyzing IDRM in a specific country will require a spatiotemporal analysis of actors, processes, and relationships that govern how disaster and risk are created/avoided, managed, and reduced in each society.

According to Alcántara-Ayala (2021, p. 329), there is a paradigm shift toward an integrative change in DRM when governance is involved, “as overarching efforts need to be strengthened first and foremost within the scope of regulatory frameworks and mechanisms of implementation.” In 2019, Alcántara-Ayala and colleagues pointed out the systematic nature of IDRM and the importance of addressing the root causes of disaster vulnerability and exposure, defining it as:

A complex *systematic* process consisting of a *series of* decisions, actions and activities, as well as a transversal coordination between the different institutional and social actors, to understand and transform the needs and weaknesses expressed in the different aspects of vulnerability and exposure, in specific responses and collective solutions, whose main objective is the deconstruction of risk. (Alcántara-Ayala et al. 2019, p. 2 own italics)

Similarly, Blümel et al. (2021) analyzed different sectors— that is, DRR, healthcare, humanitarian assistance, business management, and education—aiming at establishing a working definition of the term “integrated management.” The authors define IDRM as:

A complex and dynamic societal process in which all aspects perceived as relevant by horizontally and vertically as well as internally and externally cooperating actors are understood in their context, and corresponding effective and efficient measures are taken in a coordinated manner by all relevant actors to prevent crises or disasters and, in case of their occurrence, to avert harm and ensure the well‐being of the people at risk under dynamically changing conditions. (Blümel et al. 2021, p. 35)

Like Alcántara-Ayala (2021) and Wisner (2011), Blümel et al. (2021) underlined the “coordination” of a multiplicity of actors working on different geographical scales and at all DRM phases with a greater focus on prevention and the underlying factors of risk. One novel aspect refers to “horizontal” and “vertical” integration in contrast to “internal” and “external.” For example, in the field of science, “horizontal integration” refers to the coordination of different disciplines such as in multi-hazards research and also in transdisciplinarity. On the other hand, “vertical integration” in the public sector refers to the cooperation of different government levels—national, regional, local, and international.

Besides these, we encountered diverse cases where IDRM ideas are used in more specific domains, namely “integrated landslide risk management,” “integrated flood risk management” (IFRM), and “integrated urban risk management,” among others. Such approaches are indeed practical examples illustrating a variety of actors working on or influencing the DRM process. For example, Wang et al. (2021) studied IFRM as an effective method to reduce damage from floods in Beijing, China, while Mercado et al. (2020) studied the governance aspects of IFRM in the case of Metro Manila, Philippines. During the analysis we also detected several peer-reviewed papers and institutional reports linked to specific countries: China, Japan, and Mexico. As the literature addressing the term IDRM is in fact limited, we decided to explore these outputs. Some of the following examples reveal that IDRM ideas have moved from academic discourses to practical approaches and actual policies.

In China, according to Shi (2012, p. 140), IDRM is seen as a “basic feature of sustainable development.” Shi (2012) explored past disasters in China to point out key aspects of the Chinese government’s role in IDRM: overall leadership, engaging civil society, and international cooperation. The World Bank (2020) highlighted Shi’s observations, especially about the government’s role in overall leadership; however, it also criticized its “vertical leadership” as interests between local, provincial, and national levels in DRR rarely coincide. Shi (2012) and Zhang et al. (2004) are some of the few scholars found in our research who specifically look at IDRM in China. Unfortunately, their studies lack comprehensive reflections on how, for instance, China’s unique politico-economic system and culture influence IDRM approaches and practice.

In Japan, according to Ikeda et al. (2008), an integrated framework for DRM has been developing since the Great Hanshin-Awaji Earthquake in 1995 (at Kobe). Okada et al. (2013) illustrated these changes in Japan toward IDRM by contrasting two main DRM approaches: the *Jiishu-bosai-soshiki* and *Machizukuri*. Historically, community organizations known as *Jiishu-bosai-soshiki* (Self-support Disaster Reduction Association) were common and active after disasters. These organizations were oriented toward rescue and relief as well as self-evacuation. After the 1995 Kobe earthquake, *Jiishu-bosai-soshiki* were influenced by local governments to focus on prevention and preparedness too. In a study on *Jiishu-bosai-soshiki*, Bajek et al. (2008) concluded that they tended to be guided and mobilized by local governments, regularly supplementing government actions on reducing disaster risks in residential areas. In contrast, after the 2011 Great East Japan Earthquake disaster, the *Machizukuri* (citizen-led town-creation) approach includes a wide range of local initiatives aimed at reducing disaster risks or mitigating disaster effects. From the viewpoint of IDRM, the difference between *Jiishu-bosai-soshiki* and the *Machizukuri* approach is that the latter is holistic and multi-focused; it is therefore not limited to disaster concerns only. *Machizukuri* is citizen-led (not strongly influenced by government), involves multiple stakeholders, and takes account of day-to-day issues instead of focusing on one-time problems. Although community-led DRM has proven to be an effective way to integrate DRM at local levels in Japan, the necessary structural changes (for example, social norms and culture) to prevent the creation of disaster risks require the involvement of actors and stakeholders at all levels, such as national and regional institutions.

In Mexico, according to Alcántara-Ayala et al. (2019), the 2012 General Law on Civil Protection established the basis for an integrated management of disaster risks, defining IDRM as:

The set of actions aimed at the identification, analysis, evaluation, control, and reduction of disaster risks, considering them from their multifactorial origin and in a permanent process of construction. It involves the three levels of government, as well as all societal sectors, to facilitate the creation and implementation of public policies and strategies which are integrated to sustainable development policies and principles. These take on the structural causes of disasters and strengthen the resilience capacities of society. Integrated actions cover the stages of risk identification and/or its process of formation, forecasting, prevention, mitigation, preparation, response, recovery, and reconstruction. (Gobierno de México 2014, p. xi)

Despite this holistic statement, some studies showed that in Mexico traditional views persist (that is, vertical non-participatory governance, disciplinary silos) in DRM that rationalize the current implementation of this law (Mansilla 2019; Maurizi et al. 2020). On the other hand, Alcántara-Ayala et al. (2019) analyzed “integration” beyond DRM institutions by looking at urban development and environmental policies, budgeting, and governability processes, such as accountability. This novel angle tries to capture the comprehensiveness of DRM and DRR processes beyond the traditional and often compartmentalized DRM and civil protection domains. Interestingly, while this approach pays close attention to the interconnections and interdependencies of different public systems, the research leaves aside other relevant dimensions and sectors detected in our literature review, such as the role of academia and research institutions, civil society, and the private sector. These sectors play important roles in integrating different phases of DRM (Wisner 2011).

Based on Puente (2012) and Oliver-Smith et al. (2016), Alcántara-Ayala et al. (2019) proposed that the transition from traditional DRM to IDRM could be based on at least five normative principles: efficiency and equity, integrality, transversality, co-responsibility, and accountability. In the case of Mexico, these principles intend to guide policy design and the implementation of IDRM. Unfortunately, no recent evidence on how this approach is being taken up by Mexican authorities or recent examples of policy design were found.

The short examples from China, Japan, and Mexico show that an integrated management of disaster risks may be moving from a conceptual debate (1990–2000s) to an actual implementation debate. The examples reveal, nonetheless, that there is not a one-size-fits-all way to approach IDRM, and a mechanism or framework to assess the integration of DRM at country and city levels does not exist. The following section deepens these issues and proposes key elements to advance the study and assessment of IDRM.

## Discussion and Proposals: Proto-Indicators for Advancing Integrated Disaster Risk Management (IDRM)

The need for an integrated approach to DRM lies in the fact that the root causes of disaster risks and vulnerability are embedded in a complex web of societal processes and actors that goes far beyond the traditional domain of civil protection and risk management. This means that this integration requires looking at development processes and societal and individual relations (Erikson 1976). Thus, IDRM should not be seen only as a “part” of sustainable development pathways but as a “transversal” element without which sustainability cannot be reached.

On the importance of integrating DRR and DRM into other international agendas (that is, the Paris Agreement, SDGs, New Urban Agenda, and so on), there are three identified problems. First, if we use a systemic perspective on DRR/DRM where historically defined processes of social formation are taken into account, “sustainable development,” “disaster,” and “risk” need to be contextualized and defined in the first place, that is, from within the respective sociocultural orders one is aiming at (Voss and Dittmer 2016). This is extremely complex as it requires the development of sociocultural (sociological) methodological frameworks that orient the navigation within this complexity in different (cultural) contexts. Voss et al. (2019) are developing the conceptual Culture-and-Catastrophe Framework for this purpose. The second problem, as pointed out by Wisner (2011), refers to the relationship of disaster risk with other urgent societal issues, such as climate change, poverty reduction, gender equality, and development in general. As far as disaster risks continue being perceived as a sectoral problem, IDRM will necessarily tend to compete on different scales with other developmental issues, for both attention and resources. The third problem relates to a multi-dimensional integration. As IDRM looks to integrate with other societal agendas, it does not question if, for example, the SDGs and Paris Agreement are internally integrated themselves. This leads to the conclusion that an IDRM can exist at two levels: it can be “internally integrated” when directly related processes of disaster and risk (that is, monitoring, training, science, and so on) and related stakeholders (practitioners, decision makers, institutions, and so on) articulated at different dimensions, including spatially, temporally throughout the DRM phases, and sectorally; and it can be “externally integrated” when DRR are intertwined with other societal processes, such as gender equality, poverty reduction, and sustainable development in general. This type of integration means that DRR and DRM are coupled with multiple systems, namely, climate systems, ecosystems, and human systems (IPCC 2022). Thus, avoiding, managing, and reducing such risks entail a systemic task that goes from addressing the interactions of different coupled systems to considering the singularities of societies and territories.

There are also several challenges in implementing IDRM from a systemic point of view. As pointed out by Alcántara-Ayala (2021), little attention has been paid to integrative approaches to DRM, in part due to the resistance of compartmentalized styles of public management that have encapsulated it into rigid and often instrumentalized institutions. But beyond the changes needed for IDRM, it is necessary first to point out basic ideas that define IDRM in a general sense.

On this point, our study identified in the literature 29 elements that could lead towards an identification and assessment of IDRM (see Table 2). We have decided to call these elements “proto-indicators” as they are presented as “ideas” or “candidates” for indicators (Czúcz et al. 2021).

**Table 2:** Proto-indicators for integrated disaster risk management (IDRM)

Proto-indicators	Description	Sources
Sectoral integration (Horizontal)		
Sectoral integration	Public and private sectors, civil society, and academia work closely together and create opportunities for collaboration.	Zhang et al. (2004); UNDRR (2019, 2022a); Blümel et al. (2021)
Multi-sectoral national platforms	There are institutionalized groups (that is, platforms, roundtables) where actors from different sectors meet and discuss, and eventually collaborate in producing public policies for DRR.	Zhang et al. (2004); UNDRR (2015); Gopalakrishnan and Okada (2007)
National inter-institutional bodies	Inter-institutional bodies (inter-agency or inter-ministerial), articulating two or more institutions from different areas: for example, inter-ministerial group for disaster risk reduction.	Wisner (2011); Zhang et al. (2004); UNDRR (2015, 2019)
Resources for IDRM	Strong political commitment to promote and integrate DRR into development and climate programming: governments assign resources (financial and human).	UNISDR (2005); Wisner (2011); UNDRR (2015, 2019)
IDRM in the private sector	Integration of the private sector in disaster risk reduction efforts through promotion of business opportunities.	UN-IDNDR (1994); UNDRR (2015, 2019, 2022a)
DRR planning in the health sector	Promoting the goal of “hospitals safe from disaster” by ensuring that all new hospitals are built with a level of resilience that strengthens their capacity to remain functional during disasters.	UNISDR (2005); Blümel et al. (2021)
Critical infrastructure	Collaboration among developers and managers of critical infrastructure to avoid disruption of basic services that amplify disaster impacts.	UNISDR (2005); UNDRR (2022a)
Interdisciplinary research and science	This includes multi-hazards and interdisciplinary risk assessments.	Zhang et al. (2004); UNISDR (2005); Wisner (2011); UNDRR (2019, 2022a)
Science-policy nexus	Science for policy making and practice within DRR frameworks is developed through different mechanisms: for example, scientific evidence-based decision making takes place within governmental and legislative institutions.	Zhang et al. (2004); UNDRR (2015); Alcántara-Ayala (2021); Blümel et al. (2021); Bueb et al. (2021)
Transdisciplinary disaster risk science	Scientists and research institutions acknowledge the importance of incorporating different type of knowledge and social groups in risk assessments.	Wisner et al. (2004); Wisner (2011); Alcántara-Ayala et al. (2019)
Perspective on gender, age, disability, and cultural backgrounds	A gender, age, disability, and cultural perspective is integrated in all DRM/DRR policies and practices.	UNISDR (2005); UNDRR (2015)
Participation and ownership	DRM/DRR endeavors are subjected to active participation and appropriation by people at-risk along with their organizations.	Zhang et al. (2004); Shi (2012); Ranke (2016); Alcántara-Ayala (2021)
New divisions of labor and modes of cooperation	Development of new institutional forms (including new modes of cooperation between actors) that deal with disaster risk from a systemic approach.	Wisner (2011); Voss and Dittmer (2016)
IDRM institutionalization	Sustainable institutions and legal structures where representation of pivotal disaster risk stakeholders and the management by civil and political society are guaranteed. Here, integration, coordination, and concertation of social actors of differentiated territorial levels are “transversal” requirements for IDRM.	Zhang et al. (2004); Wisner (2011); Alcántara-Ayala (2021)
Holistic understanding of disaster risk	Understanding of disaster risk as a (dynamic and systemic) multi-dimensional phenomenon: for example, recognition that disasters are not “natural” in official documents.	Zhang et al. (2004); Wisner (2011); UNDRR (2015, 2019)
Disaster risk governance	DRM/DRR overarching efforts are strengthened first and foremost within the scope of regulatory frameworks and mechanisms of implementation.	Zhang et al. (2004); Wisner (2011); UNDRR (2015); Alcántara-Ayala et al. (2019); Alcántara-Ayala (2021)
DRR into development and planning	Integration of DRR principles into development policies and planning instruments at all government levels.	UNISDR (2005); Shi (2012); UNDRR (2015); Rosendo et al. (2018)
Synergies between DRR, SDGs, and climate change strategies	Integration of DRR into concrete SDGs and/or climate change strategies and mechanisms: for example, disaster risk-informed poverty reduction campaigns.	UNISDR (2005); Wisner (2011); UNDRR (2015, 2019); Rosendo et al. (2018); Botzen et al. (2019); Wang et al. (2021)
Spatial/hierarchical integration (vertical)		
Multi-scalar cooperation	Standards, groups, studies, or similar synergies between actors (or within) on DRM at different geographical scales or territorial levels.	Zhang et al. (2004); UNDRR (2015, 2019); Alcántara-Ayala et al. (2019)
Multi-scalar EWS	Institutional capacities ensure that early warning systems (EWS) are well integrated into governmental policy and decision-making processes (culture) as well as emergency management systems at both national and local levels.	UNISDR (2005); Shi (2012); UNDRR (2019); Alcántara-Ayala (2021)
Data integration	Integration of disaster risk-related data within national official statistics, this includes data from all DRM phases.	Zhang et al. (2004); UNDRR (2015, 2019); Lemiale et al. (2020); Blümel et al. (2021)
Scaling-up/articulation of DRR	Coordination of national DRR with international and/or major regional organizations (for example, European Union), especially with the United Nations system with emphasis on prevention.	UN-IDNDR (1994); Zhang et al. (2004); Wisner (2011); Shi (2012); UNDRR (2015, 2019)
International integration	Stronger linkages, coherence, and integration of DRM into the humanitarian assistance (both as donor and as receiver: for example, National Post-Disaster Needs Assessments). This includes efforts on supporting DRR through sustainable development assistance.	Zhang et al. (2004); UNISDR (2005); Wisner (2011)
Understanding the complexity of “territories”	Use of theories, methods, and models of analysis (quantitative/qualitative) aimed at the understanding of “territories, territoriality and habitability.” It considers their different dimensions and scales for the assessment of people’s conditions: experiences, resources, assets, capabilities, potentials, and requirements in terms of social welfare, as an inescapable—and irreplaceable—device for the reduction and management of risks.	Wisner (2011); Alcántara-Ayala (2021)
Temporal integration		
Integration across DRM phases	DRM activities and mechanism articulate throughout the DRM phases with special emphasis on prevention and preparedness.	UN-IDNDR (1994); Zhang et al. (2004); UNISDR (2005); Shi (2012)
Culture of prevention	There is a “culture of prevention” among organizations, institutions, and actors: for example, implementation of risk-informed approaches to development and planning, and avoiding disaster risk creation and impact amplification.	UN-IDNDR (1994); Zhang et al. (2004); UNISDR (2005); Wisner (2011); Shi (2012); Alcántara-Ayala (2021)
Recognition of distant root causes of disaster risks	Acknowledgment (in official documents) of the causal chain of disasters and risks that begins with distant “root causes,” spatially and temporally. This includes how disaster risks are transmitted through “dynamic pressures” such as weak government, unplanned urbanization, and so on, so shaping “unsafe conditions.”	Wisner et al. (2004); Wisner (2011); Alcántara-Ayala (2021)
Reduction of multi-dimensional vulnerabilities	Acknowledgment of the multi-dimensional nature of disaster vulnerability, as well as mechanisms to reduce social, economic, and environmental vulnerabilities to disasters.	Wisner (2011); UNDRR (2015); Alcántara-Ayala (2021)
IDRM as process (not an output)	Recognition of the dynamic and systemic nature of DRM/DRR, and its intrinsic relationship to development processes. This applies to both rural and urban systems, and it implies that IDRM is seen as a process and not as a product, while sustainability is sought throughout time.	UNISDR (2005); Ikeda et al. (2006); Shi (2012); UNDRR (2015); Alcántara-Ayala et al. (2019); Alcántara-Ayala (2021)

**Fig. 3:**
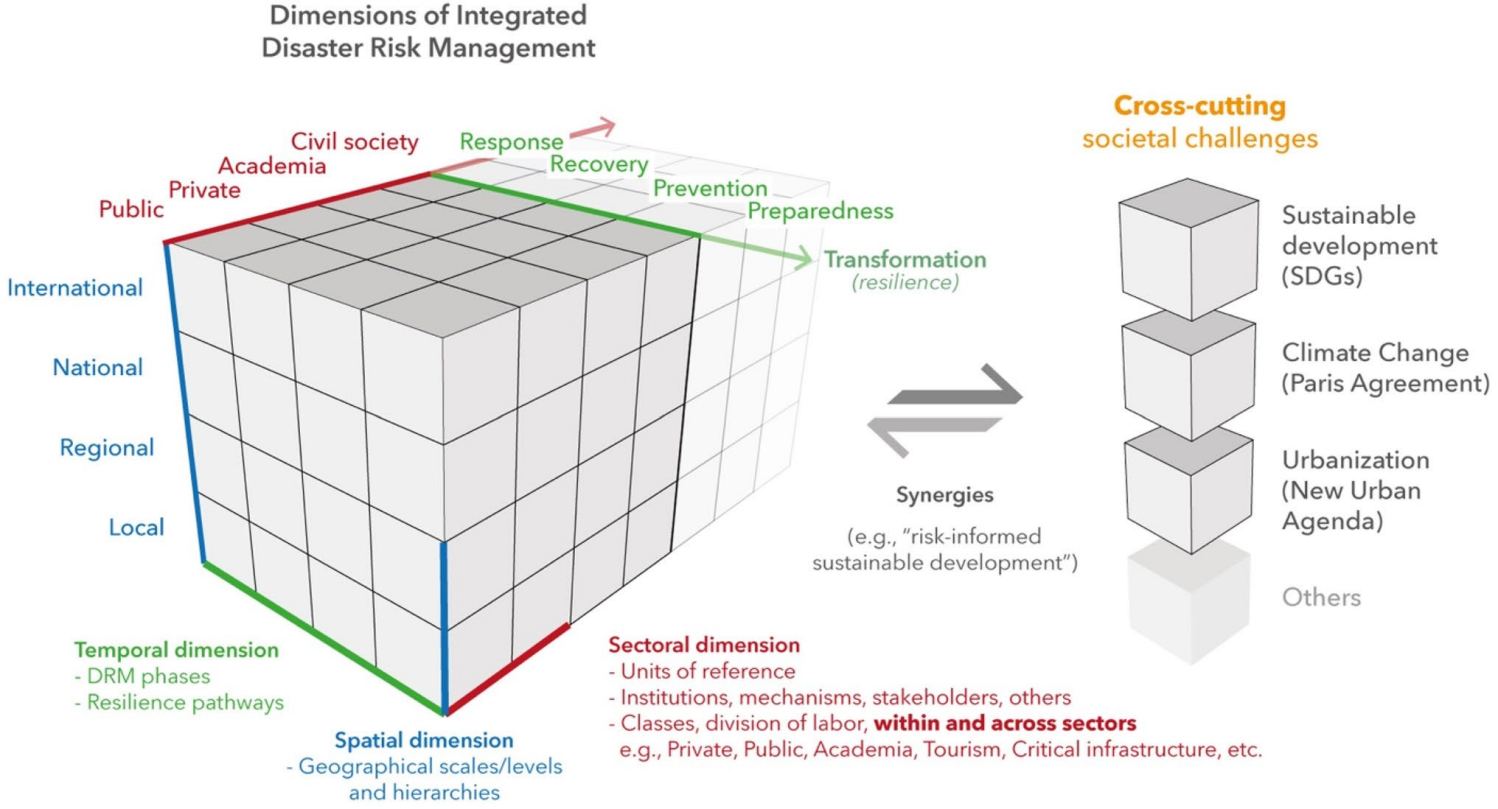
Dimensions of integrated disaster risk management (IDRM)

The 29 proto-indicators (Table 2) reflect the multi-dimensionality of IDRM in relation to at least four important dimensions or directions (see Fig. 3). First is the integration of different kinds of actors, either within (internally) or among them (externally)—for example, units of reference such as classes, groups, institutions, and social sectors. Regardless of the proposed actors in Fig. 3, the “red axe” can be expanded with other actors and institutions, depending on the specific characteristics of the context where IDRM is being analyzed. Second is integration at different geographical scales or levels, that is, actors or mechanisms of DRM that operate from the national to the local simultaneously and hierarchically. This spatial dimension (illustrated with the “blue axe”) must also include the integration of states into international and/or upper-regional levels, such as the EU Civil Protection Mechanism in Europe. Third is integration in the temporal dimension (“green axe”), that is, actors or mechanism of DRM that work throughout the main DRM phases (that is, response, recovery, prevention, and preparedness), with special focus on prevention and transformative resilience (Wisner 2011; UNDRR 2015, 2019; Asadzadeh et al. 2022). Fourth is integration of and in different relevant societal processes, such as climate change, development, and urbanization. This type of integration means that DRR/DRM thinking participates or is embedded in systemic operations of other societal processes and that it goes beyond the typical civil protection or risk management organizations. This dimension relates to the division of labor and contrasts with the traditional organization of societal sectors that have tended to work in “silos”—something recently highlighted by the Bali Agenda for Resilience in the 2022 Global Platform for Disaster Risk Reduction (UNDRR 2022b). Figure 3 uses a cube-shaped figure to illustrate the integration of DRM across different sectors, scales/hierarchies, and temporally across the DRM phases and resilience pathways (that is, transformation). Importantly, integration of DRM requires synergies with other societal challenges and processes, such as the SDGs.

## Conclusion

This study analyzed the key literature at the international level with the aim of offering a closer look at the concept of IDRM while exploring potential indicators and conceptual elements that may help to advance IDRM in national and international contexts. One of our first observations is that there is not a concrete or identifiable IDRM approach in the literature but rather a set of ideas and experiences related to what IDRM is and how it should be operationalized. Such ideas and experiences are linked to scientifically digested content and contextualized events, such as “integrated landslide disaster risk management in Mexico” (Alcántara-Ayala et al. 2019; Alcántara-Ayala 2021) or IFRM in China (Wang et al. 2021), but they are rarely based on international and comparable cases. Thus, it is possible to conclude that IDRM research still requires adopting inter- and transdisciplinary approaches that adequately incorporate other forms of knowledge and practices. In this sense, this is also a limitation of the study: analyzing experiences and elements of IDRM that are not systematically documented is a challenge for comparative and international studies, although this could be sorted out in the future with more systematization and data collection (Sarmiento and Herard 2015).

Conceptually speaking, Blümel et al.’s (2021) approach to integrated management could count as an exception. They considered IDRM multi-dimensionally and highlighted the differences between internal and external integration as well as horizontal and vertical integration. Nevertheless, Blümel et al. (2021) did not reflect on cross-cutting societal challenges, discussed in this work, in relation to the international agreements: the SDGs, the Paris Agreement, and the New Urban Agenda. Importantly, we are not here talking about “external integration” with other sectors but rather with a wide range of sectors (including their actors, stakeholders, and institutions), such as those working on climate change and sustainable development issues.

In this study we have also proposed that IDRM can be better understood from at least four dimensions: sectoral, spatial/hierarchical, temporal, and externally with other cross-cutting societal challenges. These dimensions are interconnected in a system (as we illustrated in Fig. 3), and all are necessary to achieve an effective and efficient IDRM. To that end, we proposed a series of 29 proto-indicators (or principles) to guide the exploration and analysis of IDRM in specific national contexts. Nonetheless, we have also detected the need for more comprehensive theories and methodologies to further advance IDRM in this regard.

In sum, IDRM encompasses different kinds and levels of “integrations” that go from internal (that is, DRR and DRM domains) to external (that is, all societal processes), including temporal and spatial integrations. In other words, we are talking about a dynamic and systemic integration of DRM. This makes IDRM something complex to study and especially implement, as it requires a profound understanding of the cultural and social conditions that shape DRR and DRM domains in each context. If these cultural settings are not considered or addressed in the first place, any attempt to advance IDRM (especially from an international cooperation perspective, that is, from outside) may tend to fail. Presumably, inside-out and bottom-up approaches that consider co-design and co-production of solutions and governance approaches may have greater chances of success. After all, advances in DRR and DRM are widely accessible, and knowledge is openly shared, but their usage, impacts, and effects will be diverse depending on the institutions and cultures of each society.
